# Management guidelines of penile cancer- a contemporary review of sub-Saharan Africa

**DOI:** 10.1186/s13027-020-00293-9

**Published:** 2020-05-01

**Authors:** Ayun Cassell, Bashir Yunusa, Burgess Manobah, Desire Wambo

**Affiliations:** 1 Department of Urology and Andrology, Hopital General de Grand Yoff, Dakar, Senegal; 2 Department of Surgery, Liberia College of Physicians and Surgeons, University of Liberia, Monrovia, Liberia

**Keywords:** Human papilloma virus, Penectomy, Penile Cancer, Sub-Saharan Africa

## Abstract

**Background:**

Penile cancer is a rare malignancy with prevalence higher in areas of high Human Papilloma Virus (HPV) such as Africa, Asia and South America. In middle- and low-income countries where circumcision is not routinely practiced, the rate of penile cancer could be ten times higher.

**Main body of the abstract:**

A literature review was conducted from 1992 to 2019 using PubMed, Google Scholar, African Journal Online and Google with inclusion of 27 publications with emphasis on the Sub-Saharan literature. Findings revealed that most men with penile cancer in Sub-Saharan Africa (SSA) present with locally advanced to advanced disease with devastating consequences. The option of penile sparing procedure is reduced with most treatment option directed to mutilating surgeries. The lack of appropriate chemotherapy and radiotherapy worsens the prognosis in the region.

**Short conclusion:**

Human Papilloma Virus (HPV) vaccination may not be cost-effective for most regions in SSA. Therefore, early childhood circumcision might be the best advocated alternative for prevention.

## Introduction

Penile cancer is a rare malignancy in Europe and America with an incidence of < 1/100,000 men [1]. This frequency is much higher in Africa, Asia, South America and account for up to 10% of cancer in some region. The prevalence of penile cancer is higher in nations with Human Papilloma Virus (HPV) Infection. The age adjusted incidence in Brazil is about 8.3/100,000 [2] men and was even higher in Uganda (commonest tumor in men) in the 1960s [3]. The incidence in Uganda has declined steadily probably due to urbanization, better health system and improved hygiene to a rate of 3 to 4/100,000 men [3].

The incidence of the penile cancer peaks in the sixth decade of life even though it may occur in younger patients. In middle- and low-income countries where circumcision is not routinely practiced, the rate of penile cancer could be ten times higher [4]. Conversely, in Israel where circumcision is performed exclusively for all men at birth; the incidence rate is as low as 0.1 to 0.3/100,000 men [2, 4].

The vast majority of patients in Europe and the United States will present with localized disease. This is unlikely in developing nations due to the poor knowledge of the disease, poor access to health care and low socioeconomic status. Most men with cancer in Sub-Saharan Africa (SSA) tend to present with locally advanced to advanced disease portending a dismal prognosis [5]. Penile cancer may result in distressing outcome when diagnosed lately with a five-year survival rate of 50%. When pelvic lymph nodes are involved, the 5-year survival rate may be as low as 0% [6].

This review has outlined the epidemiology and management of penile cancer in Sub-Saharan African with major highlights of the current standard of care considering the European Association of Urologists (EAU) Guideline, European Society for Medical Oncology (ESMO) and National Comprehensive Cancer Network (NCCN) Guideline on Penile Cancer.

## Methodology

The literature review was conducted from 1992 to 2019 using the various search engines and academic databases (PubMed, Google Scholar, African Journal Online and Google. Both English and French Literature search were done using the medical search heading (MEsH) and key words appended “Penile Cancer” or “Cancer Du Penis” with the following indexes: Guidelines, Sub-Saharan Africa, Liberia, Senegal, Ghana, Togo, Nigeria, Benin, Kenya, Zambia, Uganda, Zimbabwe, Tanzania. More than 200 results were found using the search terms. Only 27 articles were selected for the review based on their content either on current management of penile cancer or original article of penile cancer on the Sub-Saharan or Black African Population.

A total of ten publications of penile cancer with black African subjects were reviewed for demographics, study period, mean age, age range, occupation, comorbidities, risk factors, clinical presentation, histology, staging, management, complications and follow-up (Table 1, 2 and 3). The Tumor-Nodal-Metastasis (TNM) Staging or Jackson Staging of Penile Cancer was documented from various African studies as shown in (Table 2). Relevant data was extrapolated from the text of these publications with both quantitative and qualitative analysis conducted. The synthesized evidence was highlighted in the main text of the results and discussion sessions. The European Association of Urologists (EAU), European Society for Medical Oncology (ESMO) and National Comprehensive Cancer Network (NCCN) Guideline on Penile Cancer were considered for the discussion of penile cancer.

**Table 1:** Demographics, study period, age parameters, occupation and co-morbididty

Study	Study Period	Number of Patients	Mean Age (years)	Age Range (years)	Occupation	Co-morbidities
Ngendaho et al. Rwanda [7]	2015–2016	30	60 yrs	33 yrs–83 yrs	Petty farmers: 86.7%	HIV Infection: 20% HPV: Not tested
Diallo et al. Guinea [8]	1996–2013	06	51 yrs	32 yrs–80 yrs	N/A	HIV infection:16.7% HPV: Not tested
Magoha et al. Kenya [9]	1970–1999	55	47.9 yrs	20 yrs–80 yrs	N/A	HIV infection: not tested HPV: Not tested
Wentzel et al. South Africa (Blacks) [10]	2000–2008	65	50.9 yrs	37 yr –69 yrs	N/A	HIV Infection: 56.2% HPV: 41.5%
Gueye et al. Senegal [11]	10 years	11	55 yrs	40 yrs–75 yrs	N/A	HIV: 9.1% HPV: Not tested
Ajekigbe et al. Nigeria [12]	1990–2009	07	52.2 yrs	42 yrs–79 yrs	N/A	HIV infection: not tested HPV: Not tested
Sow et al. Cameroon [13]	1994–2005	08	N/A	43 yrs–75 yrs	Rural inhabitants: 100%	HIV infection: 12.5% HPV: Not tested
Abdulkadir et al. Nigeria [14]	1998–2015	06	59.7 yrs	50 yrs–75 yrs		
Chalya et al. Tanzania [15]	2004–2013	236	47 yrs	21 yrs–78 yrs	Unemployed	HIV infection: 6.7% HPV: 5.1%
Sow et al. Senegal [16]	2000–2011	08	51.5 yrs	27 yrs–77 yrs	Low socioeconomic status	

*HIV* Human Immunodeficiency Virus, *HPV* Human Papilloma Virus, *N/A* Not Available

**Table 2:** Risk factors, clinical presentation, histology and staging

Study	Risk Factors	Clinical Presentation	Histology	Stage (TMN/Jackson)	ILN status + Metastasis	Grade + Metastasis
Ngendaho et al. [7]	Phimosis: 50% Poor Hygiene:80% Smoking: 56.7% Lichen Sclerosis:13.3%	Subpreputial symptoms: 63.3% Penile Ulceration:26.7% Meatal lesion: 10%	SCC: 100%	T1:3.3% T2: 36.7%T3: 56.7%T4:3.3%	N0:43.3% N1:20% N2:23.3% N3:13.3% M1: 16.7%	G1:43.3% G2: 50% G3: 6.7%
Diallo et al. [8]	Late Circumcision: 66.7%	Fungating Ulcerated	SCC: 100%	T1:16.7 T2:33.3% T3:50%	N2:66.7% M1:16.7%	G1:33.3% G2:16.7% G3:50%
Magoha et al. [9]	Uncircumcision: 72.7% Late Circmcision: 21.8%	N/A	SCC:100%	Stage 1: 18.2% Stage 2: 12.7% Stage 3: 50.9% Stage 4: 18.2%	N/A	N/A
Wentzel et al. [10]	N/A	N/A	SCC: 80% VC: 15.4%	N/A	N/A	N/A
Gueye et al. [11]	Uncircumcision: 18.2% Late Circmcision: 27.3% Phimosis: 18.2%	N/A	SCC: 90.9%	Stage 1: 36.4% Stage 2: 45.5% Stage 3: 18.2%	N: 27.3% M1: 0%	N/A
Ajekigbe et al. [12]	N/A	Ulcerated Warty	SCC: 100%	N/A	N/A	N/A
Sow et al. [13]	Childhood circumcision: 100%	Ulceration Fungating Fungating + Ulcerated	SCC: 87.5% NHL: 12.5%	Stage 3&4: 100%	N2&N3: 100%	N/A
Abdulkadir et al. [14]	N/A	N/A	SCC: 100%	N/A	N/A	N/A
Chayla et al. [15]	Uncircumcision: 89.8% Smoking: 77.1% Phimosis: 62.3% Repeated STDs: 22.9%		SCC: 99.2%	Stage 1: 10.2% Stage 2: 11.2% Stage 3: 55.9% Stage 4: 12.7%	N: 65.3% M: 4.2%	
Sow et al. [16]	Poor hygiene	Fungating + Ulcerated	SCC: 100%	T1: 12.5% T2: 50% T3: 37.5% T4: 00%	N0: 62.5% N1: 00% N2: 12.5% N3: 37.5% M1: 00%	G1: 25% G2: 62.5% G3: 12.5%

*G* Grade, *M* Metastasis, *N* Node, *NHL* Non-Hodgkin Lymphoma, *N/A* Not Available, *SCC* Squamous Cell Carcinoma, *T* Tumor

**Table 3:** Management, Complication and Follow-up

*Study*	*Locality of lesion*	*Surgery*	*Chemo/Radiotherapy*	*Complication*	*Follow-up*
Ngendaho et al. [7]	Glans: 46.7%	Partial Penectomy: 80% Total Penectomy:13.3% Penectomy + ILND:33.3%	N/A	SSI:10%; Lymphocele:10%, Skin necrosis, Death, Meatal stenosis	0% recurrence at 6 months
Diallo et al. [8]	Glans: 33.3% Glans + Penile Shaft: 66.7%	PP: 16.7% TP + ILND: 16.7% Emasculation:16.7% Decline Treatment:33.3%	N/A	N/A	50% Loss to follow-up 1-year mortality 33.3%
Magoha et al. [9]	Glans: 43.6% Glans + Penile Shaft: 21.8% Prepuce: 12.7%	Circumcision: 3.6% Local Excision + Rad: 7.2% PP + Rad: 20.1% PP + Chemo: 5.5% PP + Chemo + rad: 10.9% TP + Rad: 14.5%	Rad alone: 9.1% Chemo + Rad: 10.9%	N/A	N/A
Gueye et al. [11]	Glans: 36.4% Glans + Shaft: 45.5%	Partial Penectomy: 18.2% Total Penectomy: 9.1% Decline Treatment: 72.7%	N/A	N/A	100% Loss to Follow-up
Ajekigbe et al [12]	Penile Shaft: 42.9%	N/A	N/A	N/A	N/A
Sow et al. [13]	Glans: 62.5%% Shaft: 12.5% Prepuce: 25%	TP: 37.5% Emasculation + ILND:12.5%	N/A	N/A	Loss to follow-up: 50%
Abdulkadir et al. [14]	Glans: 50% Glans + Prepuce:33.3% Shaft: 16.7%				
Chayla et al. [15]	Glans: 60.1% Glans + Shaft: 13.6% Prepuce: 7.6%	Partial penectomy: 63.1% Total Penectomy:10.8% ILND: 16.8% Penile sparing: 9.3%	Chemo: 5.9% Rad: 5.1%	SSI: 44.8%; DVT: 15.5% Chronic Pain: 13.8% Scrotal edema: 10.3%	76.1% loss to follow-up at 5 years Mortality:10% Recurrence: 5.3%
Sow et al. [16]	Glans: 12.5% Glans + Shaft: 62.5% Shaft: 25%	Partial Penectomy: 62.5% TP + ILND: 12.5% Decline Treatment: 25%			25% loss to follow-up Recurrence: 12.5% Death: 12.5%

DVT: Chemo: Chemotherapy; Deep Venous Thrombosis, ILND: Inguinal Lymph Node Dissection; N/A: Not Available; PP: Partial Penectomy; Rad: Radiotherapy; SSI: Surgical Site Infection; TP: Total Penectomy

## Results

**Fig. 1:**
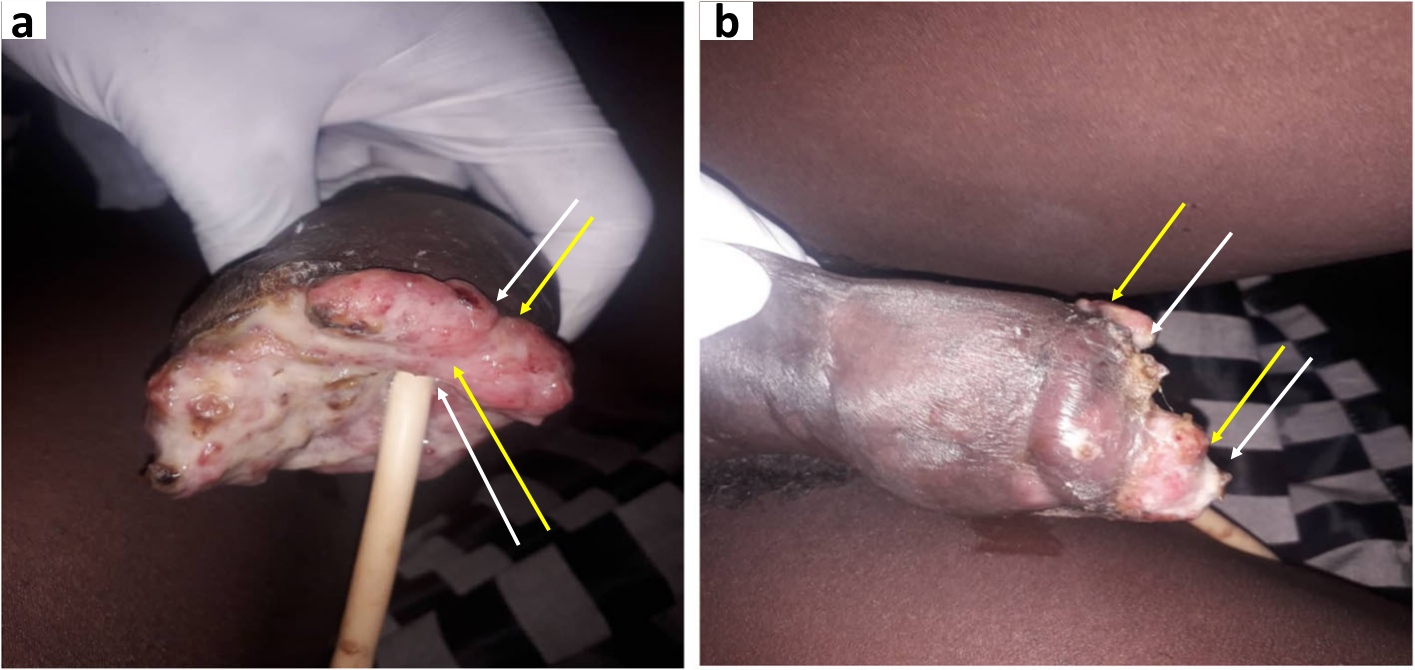
**a**-**b** Shows an advanced ulcerated and fungated penile mass that has eroded the glans, the corpora bodies and urethra

A total 10 publications [7–16] comprising of 432 black patients with penile cancer were reviewed over a period of 1970 to 2016. A pool analysis of the mean age of men with penile cancer was 52.8 years with a range of 20 years to 83 years. Most of the men in the review were petty farmers, rural inhabitants, low socioeconomic status or unemployed. Data from Guinea, Kenya, Senegal and Tanzania reported uncircumcision and late circumcision as common risk factors [8, 9, 11, 15]. Other significant risk factors in the studies were phimosis [7, 15], smoking [7, 15], poor hygiene [16] and repeated history of sexually transmitted diseases [15]. An analysis of six studies among black African with penile cancer showed the rate of concomitant Human Immunodeficiency Virus (HIV) at 20.2% (Table 1). About eight publications from the Sub-Saharan region did not test for or report the Human Papilloma Virus (HPV) amongst men with penile cancer. However retrospective study from Tanzania of 236 patients with penile cancer reported an associated HPV infection rate of 5.1%. Wentzel et al. from South Africa reviewing 65 African men with penile cancer showed a concomitant HPV infection rate of 4.5%. Most of these Sub-Saharan studies reported ulcerated, fungating, or both as the commonest presentation of men with penile cancer Fig. 1 (a-b). Squamous cell carcinoma was the predominant histological feature of men with penile cancer in the Sub-Saharan region at rate of 95.8%. Both the Tumor-Nodal-Metastasis (TNM) Staging and Jackson Staging of Penile Cancer were used (Table 2). Moreover, most men with penile cancer from the review presented predominantly with locally advanced to advanced disease. A pool analysis of 8 studies from the review showed the glans alone was commonly affected at 43.1%, glans + Penile Shaft (42.0%), Penile Shaft alone (26.8%), and Prepuce (15.1%). Reports from Rwanda, Guinea, Cameroon and Senegal revealed that partial penectomy, total penectomy + inguinal lymph node dissection, emasculation were all management options without reporting chemotherapy or radiotherapy (Table 3). However, findings from Kenya and Tanzania reported the use of adjuvant radiation therapy, adjuvant chemotherapy or adjuvant chemoradiation for men with penile cancer. An average of 43.7% of men with penile cancer from Guinea and Senegal declined penectomy. Surgical site infection, scrotal edema, lymphocele, skin necrosis, meatal stenosis, deep vein thrombosis, chronic pain were complications mentioned. Most of these men were lost to follow after a year.

### Etiology and risk factors

The Human Papilloma virus (HPV) have been identified as etiological agents associated with penile cancer. HPV Isotypes 16 and 18 have been proven to induce carcinogenesis for penile cancer. Studies from Thailand, Denmark, and Spain showed an HPV prevalence ranging from 55 to 65% [4]. Another review of 31 publication involving 1466 patients with penile cancer revealed an overall prevalence of HPV at 46.9% with dominant subtypes HPV 16 (60.2%) and HPV 18 (13.4%) [17]. Patients with Human Immunodeficient Virus (HIV) have an 8-fold increase risk of HPV infection [6] thus stipulating an essential correlation between HIV and penile cancer. Study by Wentzel et al. evaluating black South African male with penile cancer found that patients with HIV infection were more likely to develop HPV associated penile cancer at a younger age [10].

The association between the lack of circumcision and penile cancer have been well established from several studies. A meta-analysis of 7 case control studies showed a strong protective effect of childhood circumcision against invasive penile cancer (OR = 0.33; 95% CI 0.13–0.83) [18]. However, this protective effective of early circumcision was eliminated when boys with no history of phimosis were analyzed. Phimosis is a strong risk factor for invasive penile cancer with the accumulation of smegma causing repeated chronic inflammation and subsequent carcinogenesis [18]. It can be deduced that circumcision thus eliminates the risk of phimosis that could cause penile cancer. Chronic cigarette smoking, lichen sclerosis (balanitis xerotica obliterans), low socioeconomic status and education have been found as risk factors [2, 19].

The lack of circumcision as a risk in SSA remains heterogenous. Some African nations [8, 11] practice childhood circumcision as a routine ritual thus eliminating an important risk of penile cancer while others like Kenya [9] and Tanzania [15] have high rates of uncircumcised males. Despite the variation in these settings, low socioeconomic status, education and poor hygiene remain a unique challenge amongst most regions in Africa. Results from a study in Uganda showed that there was a higher rate of HPV infection from swabs of uncircumcised adult males compared to swabs taken after a year of circumcision (28% versus 18%) [4]. This data revealed that circumcision may have a protective effect against HPV infection which could portend a lower risk of penile cancer. Even with the close association of HPV infection and penile cancer, most men with penile cancer in SSA were not tested considering results from the review.

### Clinical presentation

Penile cancer may present as visible lesion along the glans, prepuce or the penile shaft. It may be associated with pain, discharge, bleeding or malodorous based on the stage of the disease. The disease presentation can be described as nodular, ulcerative or fungating [6]. In some cases, penile cancer can be concealed by phimosis. In advanced cases of penile cancer, constitutional symptoms (fatigue, weight loss) and palpable inguinal lymph nodes could be present [6]. Most men with penile disease in SSA present with advanced stages of penile cancer either due to lack of knowledge, socioeconomic status or fear of the treatment options. Therefore, common clinical presentation reported from Rwanda [7], Guinea [8], Nigeria [12], Cameroon [13], and Senegal [16] included penile ulceration, fungating mass or ulcero-fungating mass Fig. 1 (a-b). These clinical descriptions denote a late presentation of the disease.

### Pathology

Squamous cell carcinoma (SCC) is the predominant histological type in 90% of penile cancer. SCC of the penis can be further categorized as basaloid, verrucous and papillary types [2, 19]. Less common histological variants include adenocarcinoma, melanoma and sarcoma. The growth pattern could be superficial spreading, nodular, vertical growth or verrucous growth [1]. Race and geographical variations did not affect much the histological type of penile cancer as this review displayed SCC the commonest among SSA males with an average of 95.8%.

### Diagnosis and staging

The diagnosis of penile cancer is confirmed by biopsy. Pathological report is essential to drive treatment plan and lymph node management. Excisional biopsy is ideal to assess the degree of invasion and stage of the disease. Intraoperative fresh frozen section can depict negative surgical margin; 5 mm of tumor free margin is considered oncologically adequate [2]. Perform physical exam and document the number of nodes, morphology and laterality. If inguinal lymph nodes are not palpable, offer invasive lymph node staging for only high-risk patients. Otherwise, imaging (Ultrasound, Magnetic Resonance Imaging (MRI) Computed Tomography (CT) scan) may not be helpful to detect micro metastasis. Patient with palpable inguinal lymph node may require pelvic, abdominal and thoracic CT-scan to assess pelvic lymph nodes and distant metastasis [1, 2].

The American Joint Committee on Cancer (AJCC) tumor, nodes and metastases (TNM) classification has staged penile cancer for prognostic purposes. Stage 0 (Tis, Ta, N0, M0: non-invasive verrucous carcinoma or carcinoma in situ) has a 90–100% five-year survival rate [19]. Similarly, Stage 1 (T1a, N0, M0: with no evidence of lympho-vascular invasion LVI) has a 5-year overall survival of between 90 and 100%. Stage 2 (T1b/T2/T3, N0, M0: LVI/ undifferentiated/poorly differentiated/tumor invades corpus cavernosa/ corpora spongiosum or urethra) also has a good prognosis after treatment [19]. Stage 3A (T1–3, N1, M0) single, unilateral lymph node has an overall 5-year survival (OS) rate at 80%. Contrarily, Stage 3B (T1–3, N2, M0) multiple unilateral or bilateral inguinal lymph nodes has a 5-year OS of 40%. More advanced Stage 4 (any T4, any N3, any M1) T4 – invades contiguous structures, N3 – fixed inguinal or pelvic lymph nodes, or metastatic disease has a dismal 5-year OS at 11% [19]. According to the AJCC, SCC of the penis is graded based on the degree of cell anaplasia [20]. Grade-1 is considered well differentiated, grade-2 is moderately differentiated, grade-3 is poorly differentiated while grade-4 is undifferentiated [20].

Like most other late cancer presentation in SSA [5], most patient in the review presented with locally advanced to advanced penile cancer. In the absence of relevant education, men in SSA see it as a taboo to communicate about genital problems. There is also a misconception that every penile lesion may lead to total penectomy or emasculation if reported to a health facility. The unfortunate paradox is that this fear and misconception leads to very late presentation amongst SSA men with penile cancer actually necessitating these mutilating procedures.

### Recommended management guideline

#### Tis/Ta

Patients with non-invasive verrucous carcinoma or penile carcinoma in situ can benefit from penile preserving modalities including topical 5-fluorouracil or 5% topical imiquimod [1, 2, 6]. Circumcision, wide local excision (Mohs procedure), partial/complete glansectomy as well as laser therapy (carbon dioxide or neodynium:yttrium-aluminum-garnet) have shown good results [1, 2, 6].

#### T1/G1–2

Penile preserving procedures should be carefully selected in patients regarding the ability for follow-up. Studies have shown that the 2-year recurrence rate may approach 50% [11]. Circumcision, wide local excision (Mohs procedure), partial/complete glansectomy, laser therapy or radiotherapy (external beam or brachytherapy) have been indicated as treatment options. Current evidence has shown that a surgical margin of 5 mm possesses similar oncological safety as 2-cm margin [6].

#### T1/G3–4, T ≥ 2

Partial or total penectomy will be require for these lesions considering the characteristics of the tumor and depth of invasion. Intraoperative fresh frozen section should be used to attain negative surgical margin. For select patients with tumor occupying less than half of the glans, wide local excision or glansectomy are options if the patients can adhere to strict follow-up plans [6]. The possibility of repeat wide local excision for local recurrence without cavernosa invasion or partial/total penectomy for a more invasive disease should be discussed with the patient. For lesion less than 4 cm, radiotherapy by external beam or as brachytherapy are treatment option [2, 6].

The management of invasive disease confined to the corpus spongiosum or glans (T2) include total glansectomy with reconstruction of the corporal head. The treatment of disease involving the corpora cavernosa and/or urethra (T2/T3) include partial penectomy with reconstruction [2, 21]. Radiotherapy should be considered as an alternative but will require circumcision first. Patient with locally advanced (T3) penile cancer should be treated with total penectomy with a perineal urethrostomy. More advanced (T4) disease should require neoadjuvant chemotherapy followed by surgery when response is observed [2, 21]. However, adjuvant chemotherapy or palliative radiation are alternatives.

### Management of Lymph Nodes

#### Nonpalpable lymph nodes

Clinically negative node patients with low risk (Tis, Ta, T1G1) and intermediate risk (T1G2) can undergo surveillance. However, patients with nonpalpable inguinal lymph node with intermediate risk and high-risk penile cancer dynamic sentinel node biopsy (DSNB) is recommended [1, 2, 6]. Inguinal lymph node dissection is recommended if a positive node is identified.

#### Unilateral or bilateral palpable inguinal nodes [1, 2, 6]

For patients with palpable lymph node, a Fine needle aspiration is recommended. If results are equivocal, an excisional biopsy is indicated to confirm. In the presence of a positive lymph node, an immediate inguinal lymph node dissection should be performed. When greater than two nodes are positive or extranodal disease, a pelvic lymph node dissection is recommended. Adjuvant chemotherapy should also be considered.

#### Fixed or ulcerated inguinal lymph node [1, 2, 6]

The risk of metastasis is very high in these patients therefore, pelvic, abdominal CT-scan is indicated. Multimodal treatment with neoadjuvant chemotherapy with subsequent radical lymph node dissection in those with clinical nodal response.

#### Recurrent regional lymph node [1, 2, 6]

There is no level evidence for the ideal management of recurrent nodal disease. However, multimodal treatment including radical lymph node dissection, chemotherapy or radiotherapy are options.

#### Metastatic penile cancer

Systemic chemotherapy, radiotherapy or chemoradiation should be considered for patients with metastatic disease. The first line systemic chemotherapy regimen is cisplatin based (paclitaxel, ifosfomide + cisplatin or 5 Fluorouracil + cisplatin) [2, 6]. Radiotherapy may be used for palliation. Immunotherapy like pembrolizumab can be used as a second line. Advanced case of penile cancer refractory to systemic therapy tend to rely on supportive care. The mean 5-year overall survival for metastatic penile cancer is about 10% [6, 22].

Considering the late presentation of penile cancer from this review, more mutilating procedures were required compare to penile preserving surgeries. Data from Guinea and Senegal showed a high rate of decline from surgeries after surgeons suggested penectomy as a treatment plan. The use of chemotherapy or radiotherapy was barely reported except for data from Kenya and Tanzania that used adjuvant chemotherapy or radiotherapy in few patients [9, 15]. Most of the region lack cancer treatment centers as well urologist, oncologist or radiation oncologist. Radiotherapy is sparsely distributed on the African Continent with South Africa and Egypt have the most centers [23]. The World Health Organization (WHO) declared that there are only 22 chemotherapeutic agents available to Africa at varying time, but these drugs are rather too expensive for an average person from SSA [23]. This mishap is worsened by the population at risk for penile cancer; those of low socioeconomic status that cannot afford treatment.

### Follow-up

The goal of follow-up is to detect early recurrences that could be curable and the management of treatment related complication. A more intense follow-up regimen is recommended during the first 2 years where recurrence is expected to be high. A less rigid follow-up plan can continue up to 5 years; thereafter which patient can carry out self-examination at home [1, 2, 6].

The follow-up in the review was dismal amongst men managed for penile cancer in SSA ranging from 25% to 100 loss to follow-up. Most patients are psychologically and emotionally challenged after penile procedures and may not return to the health institution knowing that their penile length will not be restored. The cost of treatment, distance from major cancer center and unreported death at home may be contributing factors.

### Quality of life after penile procedures

Evidence has shown that there is acceptable sexual activity and quality of life after penile sparing procedures like laser surgery or wide local excision. However, some decline in sexual activities have been observed after glansectomy and more after partial penectomy [2, 24]. The challenge has been the length of the penile stump may not be suitable for satisfactory sexual intercourse [2, 24]. Some patients have reported to loss self-esteem as well as sexual pleasure. A strong family support system should be established before proceeding to penile procedures. The psychological well-being of the patients as well as the partners have a crucial role to play in the follow-up process.

### Prevention strategies and recommendation

Neonatal circumcision as practiced by the Jews has seem to mitigate the risk of phimosis thus reducing the incidence of penile in this population [2, 6, 15]. Some regions are now suggesting routine neonatal circumcision as means to reduce the risk of penile cancer. Increasing daily hygiene and preventing sexually transmitted infection could be helpful in alleviating the risk of penile cancer [25]. The HPV vaccine has been shown to the reduce the transmission of HPV infection thus reducing the risk of cervical cancer in female and precancerous anal lesions in men [26]. It is postulated that the HPV vaccine could provide some protection against penile cancer in men by preventing the transmission of HPV infection. There is still some skepticism about the cost effectiveness of HPV vaccination while others are concerned that it may increase male promiscuity. However, in the context of SSA, neonatal circumcision seems the most cost-effective option.

## Conclusion

Penile cancer is rare with SCC being the commonest histological type. HPV, lack of circumcision, phimosis, poor hygiene and low socioeconomic status are established risk factors. Most men with penile cancer in SSA present with locally advanced to advanced disease with devastating consequences. The option of penile sparing procedure is reduced with most resulting to mutilating surgeries. The lack of appropriate chemotherapy and radiotherapy worsens the prognosis in the region. HPV vaccination may not be cost-effective for most regions in SSA therefore, neonatal circumcision might be the best advocated alternative for prevention.

## Data Availability

Not applicable.

## Abbreviations

HIV: Human Immunodeficient Virus
HPV: Human Papilloma Virus
SSA: Sub-Saharan Africa
TNM: Tumor-Nodal-Metastasis
WHO: World Health Organization
